# Overcoming Imaging Limitations: 4D‐STEM Pair Distribution Function for Resolving Structure of Supported Pt_55_ and Pt_200_ Clusters

**DOI:** 10.1002/smtd.71003

**Published:** 2026-08-28

**Authors:** Ajai Raj Lakshmi Nilayam, Ramin Shadkam, Sangjun Kang, Horst Hahn, Di Wang, Christian Kübel

**Affiliations:** ^1^ Institute of Nanotechnology Karlsruhe Institute of Technology Karlsruhe Germany; ^2^ Department of Materials and Earth Sciences Technical University of Darmstadt (TUD) Darmstadt Germany; ^3^ Karlsruhe Nano Micro Facility (KNMFi) Karlsruhe Institute of Technology (KIT) Eggenstein‐Leopoldshafen Germany; ^4^ Department of Materials Science and Engineering University of Arizona Tucson Arizona USA

**Keywords:** bond length, coordination isomerism, electron diffraction, metal clusters, pair distribution function, scanning transmission electron microscopy

## Abstract

Metallic clusters in the 2–200 atom range exhibit exceptional functional properties arising from their large fraction of under‐coordinated surface sites and quantum size effects. However, structural characterization of these clusters is challenging due to the absence of well‐defined periodicity and long‐range crystal structure. In this size regime, surface and finite‐size effects dominate, leading to highly relaxed and/or multiply twinned configurations minimizing total energy. Experimental approaches using x‐rays and electrons require particular care, as irradiation can distort the intrinsic structure of these clusters. To accurately resolve their structure, probing techniques must operate at sufficiently low dose to avoid atomic displacement from equilibrium sites. Here, we investigate the structure of Pt_55_/Pt_200_ clusters supported on amorphous Carbon from room temperature to catalytically relevant conditions using 4D‐STEM‐based pair distribution function (PDF) analysis, demonstrating its power to resolve intrinsic atomic arrangements. The atomic structures of Pt_55_/Pt_200_ clusters differ significantly from bulk Pt, exhibiting mixed characteristics of ideal isomeric configurations combined with contributions from chemical bonding to the substrate. The temperature dependence of the Pt–Pt bond length, measured from room temperature to 500°C, is used to extract the linear thermal expansion coefficient (TEC), which exhibits a pronounced enhancement of TEC with size confinement.

## Graphical abstract

4D‐STEM based electron diffraction analysis has been developed to characterize the average structure of sensitive, dynamic ultrasmall supported Pt clusters at variable temperature by pair distribution analysis (PDF). For Pt_55_ and Pt_200_ the best fit is obtained for a multiply twinned decahedral configuration. Illustration prepared by ChatGPT based on manuscript.

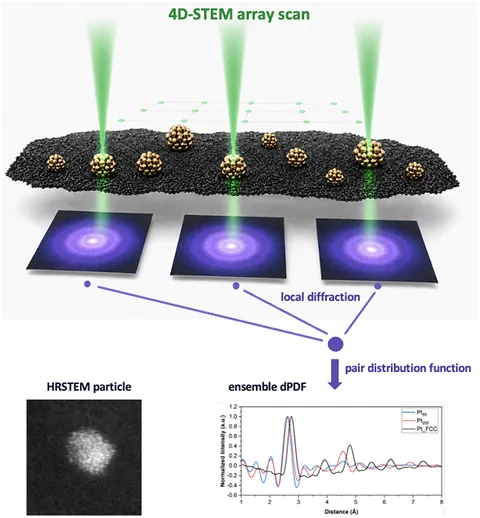

## 1 Introduction

Small metallic clusters (from 2–200 atoms) occupy a unique size regime between isolated atoms and bulk materials. In this regime, properties vary dramatically with size, composition, and atomic structure, enabling a broad range of applications including heterogeneous catalysis [1, 2, 3, 4, 5], optical and electronic devices [6, 7, 8, 9], bioimaging, sensing and drug delivery [10, 11, 12, 13], energy conversion and storage [14, 15, 16, 17], and quantum technologies [18, 19]. This versatility arises from their low coordination numbers and high surface‐to‐volume ratio, which lead to modified electronic structures resulting in numerous “active sites.” Consequently, precise structural characterization of clusters is essential to understand the origin of their unusual properties and to guide their rational for specific applications.

The structure of atomic clusters in the size range of a few to several tens of atoms has been extensively studied in the past, predominantly using theoretical approaches. These include density functional theory (DFT), Monte Carlo, and molecular dynamics simulations, often combined with global optimization strategies such as genetic algorithms to identify thermodynamic equilibrium structures or low‐potential‐energy isomers. In practice, the computational cost of fully first‐principles DFT calculations increases rapidly with cluster size, particularly when many candidate isomers, charge states, spin states, support interactions, or finite‐temperature effects need to be considered. Full ab‐initio DFT studies are typically limited to relatively small clusters, generally up to a few tens of atoms (∼100 atoms), owing to their high computational cost, whereas larger sizes are more commonly explored using classical methods. Complementary to these theoretical efforts, sophisticated experimental techniques such as trapped ion electron diffraction (TIED) enable the preparation and structural characterization of charged clusters (Ag, Au, Ni, Pt, Pd, etc.) in the equilibrated gas phase [20, 21, 22, 23]. Depending on the atomic species, TIED experiments can access cluster sizes ranging from a few atoms up to roughly 100–150 atoms, thereby providing a critical experimental reference for theoretical predictions. For example, using TIED combined with DFT including spin–orbit coupling, Bumüller et al. resolved the size‐dependent structural evolution of platinum cluster anions Pt_n_^−^ (*n* = 6–13). The clusters evolve from planar and amorphous‐like motifs to tetrahedral and finally hcp‐based structures, with several sizes exhibiting coexistence of multiple isomers. The experimentally observed TIED patterns often differ from the DFT‐predicted global minima, indicating that reliable structural assignment requires consideration of the broader structural ensemble and the specific experimental conditions. Furthermore, the cluster size regime accessible through this combined theoretical and experimental approach still lies below the metal nanoparticles size typically employed in heterogeneous catalysis. Importantly, it should be noted that TIED probes charged gas phase cluster, whereas the structure of neutral clusters can differ substantially, especially after deposition on a substrate. In such supported systems, metal‐substrate interactions play a crucial role and must be explicitly considered. This substantially complicates theoretical modeling, as the thermodynamically favored gas‐phase structure may no longer be preserved and additional effects such as structural distortion and cluster flattening induced by the substrate need to be accounted for. A detailed understanding of the native chemical state and atomic structure of these clusters is therefore essential for gaining insight into the properties and performance of supported catalysts. This, in turn, highlights the need for advanced experimental techniques capable of directly probing cluster structures under well‐defined conditions closer to application.

Scanning transmission electron microscopy (STEM) is extensively used to observe small (size‐selected) clusters to visualize the crystal structure and to compare the observed projection images to theoretically calculated structural models [24]. A conventional approach for beam‐sensitive metallic cluster assemblies in Annular Dark Field‐STEM (ADF‐STEM) is to acquire time‐resolved image series, recover interpretable atomic contrast by spatiotemporal denoising, constructing candidate 3D atomic models from atom‐counting across multiple projected views, and validate these structures by comparison with frozen‐phonon multi‐slice image simulations [25]. At the same time, the electron‐beam can strongly perturb sub‐nanometer clusters by driving diffusion, rotation, restructuring, and crystallization or disordering, so that the observed configurations must be interpreted as the combined outcome of temperature and beam‐induced changes. Thus, with reduced size and depending on the element and chemical environment, imaging with electrons faces severe limitations due to the energy transfer from the high energy electron‐beam, resulting in permanent variations of the structure (knock‐on damage, radiolysis) [26, 27]. Even when imaging below the knock‐on damage thresholds for bulk materials, electron‐beam exposure during high‐resolution imaging can still induce substantial structural changes in cluster assemblies. Because the energy differences between competing cluster configurations are small in the sub‐eV to few eV range [20, 24], clusters can already undergo rapid thermally activated structural fluctuations and the energy transferred by the electron‐beam can induce further rearrangements beyond thermal activation giving rise to new configurations. This may also induce sputtering of low‐coordination surface atoms and trigger diffusion and support‐mediated mobility that can lead to cluster ripening or aggregation.

In addition to electron microscopy, x‐ray‐based characterization techniques are also widespread in elucidating the structure of cluster‐assembled materials. These methods provide complementary averaged structural information even under realistic working conditions with in‐situ and/or operando. However, a conventional laboratory scale x‐ray diffraction is generally not suitable, due to the small size of the clusters and the absence of long‐range crystalline order. The resulting diffraction signals are either too broad or too weak to render reliable structural determination. Hence, grazing incidence x‐ray scattering at wide and small angles (GIWAXS and GISAXS) are utilized together to conduct real‐time analysis and determine the structure of bulk samples of cluster ensembles but unambiguous structural determination of ultra‐small clusters (below 1.5 nm in diameter) is challenging with x‐ray scattering due to the dominance of surface disordering at such small scales [28]. In contrast, synchrotron‐based techniques, particularly x‐ray absorption spectroscopy (XAS) with extended x‐ray absorption fine structure (EXAFS), are widely used and capable of delivering average coordination numbers and bond lengths of the dominant species [29, 30]. Despite these advantages, EXAFS also has inherent limitations. As an averaging technique, it provides only ensemble‐averaged structural information and is therefore insensitive to minority species or structural heterogeneity within the sample.

Diffraction‐based atomic pair distribution function (PDF) analysis has emerged as a powerful tool for structural characterization across multiple length scales in recent years [31, 32]. By providing real‐space information from the local atomic environment to nanometer‐scale ordering, PDF analysis bridges the gap between conventional EXAFS, which is typically limited to short interatomic distances, and Bragg diffraction, which is sensitive primarily to long‐range periodic order (>3 nm) [33]. Synchrotron‐based x‐ray PDF measurements are widely employed and are applicable to a broad range of materials; the large accessible momentum transfer, often exceeding 30 Å^−^^1^, enables high real‐space resolution. However, the relatively weak scattering cross‐section of x‐rays necessitates comparatively large sample volumes. Electron‐based PDF (ePDF), by contrast, benefits from the much stronger interaction between electrons and matter and can be readily performed using standard transmission electron microscopes, allowing for high‐quality PDF data to be collected from very small quantities of material and even from spatially selected regions [34]. PDF acquisition using four‐dimensional scanning transmission electron microscopy (4D‐STEM) provides exceptional flexibility, with tunable spatial resolution from one nanometer to several nanometers by changing the incident electron probe size, with spatially resolved PDF mapping capabilities [35, 36, 37]. Atomic PDF analysis is well suited for the characterization of supported metal clusters as it allows working with electron doses several orders less than the converged electron probe used for conventional atomic resolution STEM imaging, making it dose efficient and reliable for probing the native atomic arrangement of electron‐beam sensitive specimens.

In this work, we investigate Pt_55_ and Pt_200_ clusters deposited on amorphous Si_3_N_4_ and amorphous carbon substrates to elucidate their stability, structural evolution, and cluster–support interactions under electron‐beam irradiation and at elevated temperatures. By combining in‐situ heating experiments with 4D‐STEM‐based PDF analysis, we assess the cluster stability and resolve the atomic‐scale structure and bonding environment of supported clusters. This approach enables direct quantification of Pt–Pt bond lengths, Pt surface oxidation contributions, identification of Pt–C interactions, and determination of size‐dependent thermal expansion behavior. The results provide fundamental insight into the role of support chemistry and size confinement in governing the stability and atomic structure of supported Pt clusters, while demonstrating the unique capability of 4D‐STEM PDF analysis to probe both intra‐cluster structure and cluster‐support interactions with atomic‐scale sensitivity.

## 2 Results and Discussions

**FIGURE 1:**
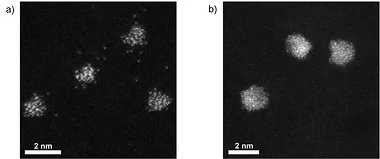
HRSTEM image of (a) Pt_55_ clusters on amorphous C, (b) Pt_200_ on amorphous Si_3_N_4_.

High‐resolution STEM images of supported Pt_55_ and Pt_200_ clusters are presented in Figure 1a,b. For both cluster sizes, well‐defined atomic columns, commonly observed in larger faceted nanoparticles are absent, reflecting the insufficient number of atoms to form stable, fully faceted geometries in these ultrasmall clusters, where multiple isomeric configurations with similar energies are expected. The thermal fluctuations and instability under the electron‐beam of Pt_55_ and Pt_200_ clusters are clearly demonstrated by the HRSTEM time‐series images shown in the Supporting Information Figure S1 and S2. The observed mobility and weak adhesion of the clusters on Si_3_N_4_ likely arise from the combined effects of thermal energy at room temperature and electron‐beam irradiation. Under these conditions, clusters are seen to continuously change shape, exchange atoms, and, in some instances, coalesce with neighboring clusters. This is also accompanied with the STEM imaging induced C‐contamination of the imaging area, which is inevitable and hinders the high‐resolution Z‐contrast imaging.

**FIGURE 2:**
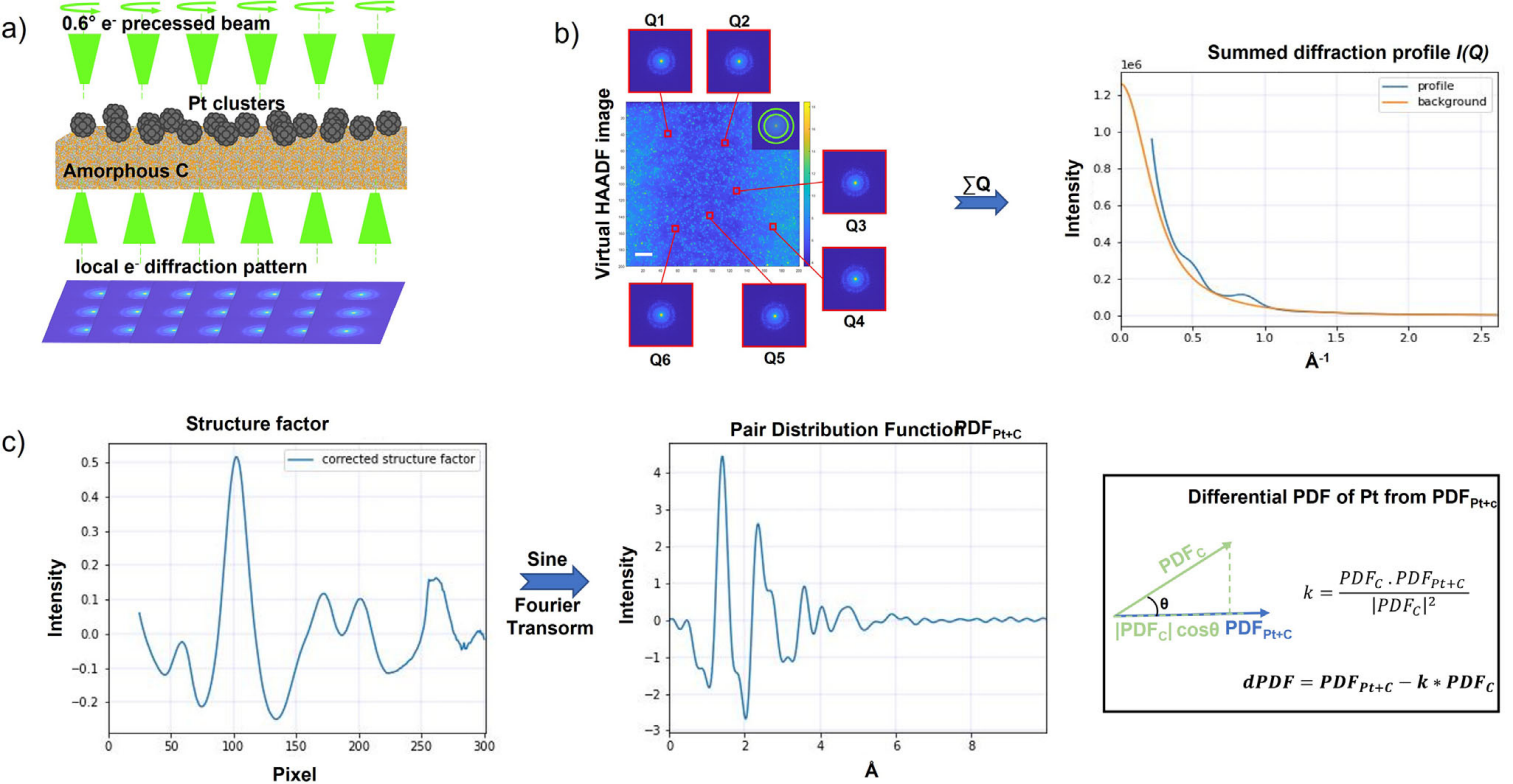
(a) Schematic of 4D‐STEM data acquisition with 0.6° precession angle. (b) Identification of the Pt clusters based on the virtual HAADF image intensity (scale bar is 20 nm) after C‐background polynomial fitting. (c) The integrated and radially averaged diffraction profiles were processed by subtracting the single atomic scattering factor to obtain the average PDF_Pt+C_ (Pt together with C underneath). The C contribution to the PDF was removed by weighted subtraction of PDF_C_ from the PDF_Pt+C_ to obtain the averaged dPDF of the pure clusters.

To overcome the limitations of direct high‐resolution imaging, we used 4D‐STEM‐based PDF analysis. With a 0.6° precession probe, this approach enabled probing of the native structure of Pt_55_ and Pt_200_ at room temperature and tracking structural changes at elevated temperatures using clusters on MEMS heating chips for in‐situ TEM. The data collection and analysis process are detailed in Figure 2. The Pt clusters supported on the amorphous carbon membrane of the MEMS heating chip are randomly oriented and randomly distributed. The Pt clusters were identified in the 4D‐STEM dataset using the HAADF scattering intensity extracted from the 4D‐STEM diffraction data. A challenge was separating the Pt signal from carbon contamination when scanning at room temperature. The contamination leads to smooth variations in the background intensity across the HAADF image, typically varying significantly from the edges toward the center, as can be seen in Figure 2b. To correct the HAADF intensities for the contamination, a polynomial background fitting was applied to the HAADF intensities along both the x and y directions. This procedure removes the gradual intensity variations and produces a uniform background across the field of view, allowing reliable extraction of the cluster signal from the HAADF intensity (for details, see experimental section). Agglomerated clusters were excluded from the 4D‐STEM dataset prior to PDF analysis to ensure that only isolated cluster signals are considered. The extracted diffraction patterns for the Pt clusters, together with the underlying carbon contribution, were summed and radially integrated to obtain the average structural information of the supported clusters (Figure 2b). The single atomic scattering factor was removed from the diffraction intensity to obtain the reduced scattering function, which was subsequently subjected to a sine Fourier transform to generate the real‐space PDF following the procedures outlined by X. Mu et al. [35]. and S. Kang et al. [38]. To isolate the Pt structural signal from the averaged PDF_Pt+C_, the carbon contribution needs to be subtracted. This was achieved by extracting the PDF from carbon‐only regions in the 4D‐STEM dataset to obtain the pure carbon PDF (*PDF_C_*). A weighted subtraction of the *PDF_C_* from the averaged PDF_Pt+C_ was used to obtain the pure Pt_55_ and Pt_200_ differential PDF (*dPDF*), as shown in Figure 2c. The complete procedure, from extraction of the Pt cluster signal to the final carbon background subtraction, is described in detail in the Experimental Section. A comparison of PDF_Pt+C_, PDF_C_ and the Pt_200_ dPDF obtained after the weighted carbon background subtraction is shown in Figure S4. The subtraction effectively suppresses not only the characteristic C–C bond at 1.43 Å, which serves as the primary fingerprint of C, but also significantly reduces the longer‐range carbon‐carbon correlations, thereby isolating the Pt‐related structural features.

**FIGURE 3:**
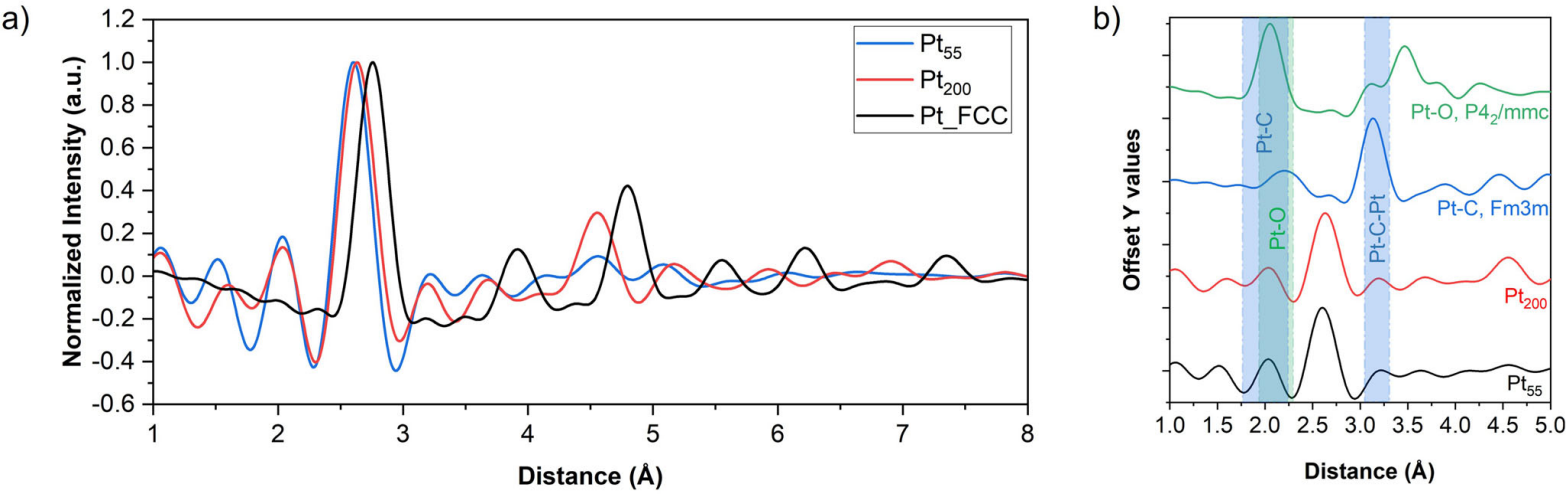
(a) Comparison of Pt_55_ and Pt_200_ dPDF with simulated FCC‐Pt PDF. (b) Pt–O, Pt–C and Pt–C–Pt bond lengths in Pt_55_ and Pt_200_ compared with references PtC Fm3̅m PDF and PtO P4_2_/mmc PDF. The shaded region highlights the range of reported possible bond lengths for Pt–O, Pt–C and Pt–C–Pt, accounting for variations in local coordination geometries and arrangement of O and/or C around Pt, other than the references shown in the comparison.

**FIGURE 4:**
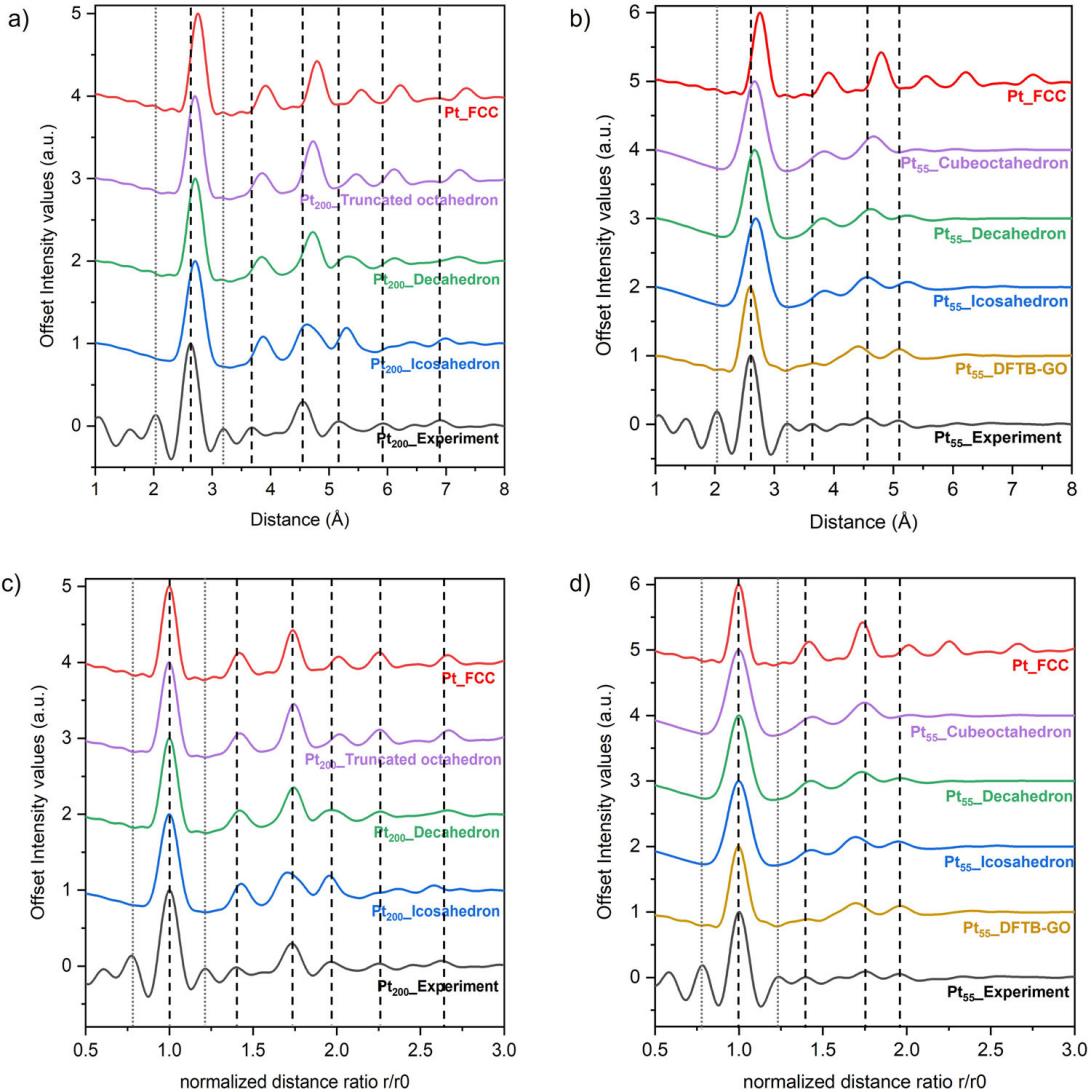
Experimental dPDFs of (a) Pt_200_ and (b) Pt_55_ compared with the corresponding simulated relaxed icosahedral, decahedral, cubeoctahedral/ truncated octahedral, and bulk FCC Pt structures. Panels (c) and (d) show the same dPDF data for Pt_200_​ and Pt_55_​, respectively, with the distances represented in normalized form as r/r0, where r0 is the average Pt–Pt bond distance of the experimental dPDF. Here the x‐axis shows the normalized distance ratio r/r0 ​, such that the average Pt–Pt bond distance corresponds to r/r0 = 1.

The dPDF obtained for Pt_55_ and Pt_200_ are compared with FCC‐Pt in Figure 3a. The observed Pt‐Pt bond distance from the dPDF is 2.60 Å for Pt_55_ and 2.63 Å for Pt_200_, which is contracted by ∼6% and ∼5%, respectively, compared to the bulk Pt–Pt bond distance (2.77 Å) (Table S1). This change in bond distance can be attributed to finite‐size effects in the cluster: surface relaxation and strain due to undercoordinated surface atoms in these small clusters lead to a contraction of the average bond length as reported previously in other cluster systems [39, 40]. The slightly larger average bond length in Pt_200_ fits to this cluster being larger in size, slightly closer to the bulk. The characteristic distances for the second and higher order coordination shells in Pt_200_ and Pt_55_ are both shifted, and their intensities are noticeably reduced compared with the bulk Pt, in agreement with a reduced medium/long range order in the clusters. This is particularly noticeable for Pt_55_, where no correlations beyond 6–7 Å are visible in the dPDF fitting to the particle size of around 8 Å. In addition, a closer inspection of the relative position of the higher order correlation distances versus the Pt–Pt bond length (Figure 4; Table S1) indicates that the local symmetry has changed in the clusters. This might be partially due to size effects, as will be discussed below, and is partially also triggered by the interaction with the carbon support film, which fits to two additional characteristic distances of 2.04 Å for Pt–C bonds and 3.21 Å for Pt–C–Pt bonding [41, 42], which are present in both Pt_55_ and Pt_200_. In addition, Pt–O bonding cannot be excluded as the Pt–O bond length [43, 44] is similar to the Pt–C bond length, so that a clear distinction between both is not possible considering that slight shifts might be introduced by the subtraction of the carbon background. Moreover, a range of bond distances is reported for Pt–C and Pt–O because the surface Pt atoms experience different local coordination environments and oxidation states, undercoordinated and less oxidized Pt sites generally form shorter Pt–C bonds, whereas more oxidized Pt sites preferentially form shorter Pt–O bonds due to stronger Pt–O interactions [42, 45, 46].

For a more detailed analysis of the structural variations induced in the small clusters compared to the bulk, Figure 4 presents the dPDFs of Pt_200_ and Pt_55_ measured at room temperature alongside modeled PDFs of various isomeric candidate structures for the clusters based on icosahedral, decahedral, and cubeoctahedral/ truncated octahedral Pt clusters relaxed with the EMT potential implemented in ASE (see Supporting Information for details). As expected, the average Pt–Pt bond length of 2.67 to 2.71 Å in these model structures is reduced compared to bulk Pt, but it is still noticeably larger than the experimentally measured bond length in Pt_55_ and Pt_200_. This difference is most likely due to the relatively low‐level EMT potential employed in the ASE package. For Pt_55_, van den Bossche et al. [47] published a structure obtained using a global optimization workflow based on DFT level calculations (DFTB‐GO), which is in good agreement with the Pt–Pt bond length in our experimental dPDF data. The reported DFTG‐GO Pt_55_ cluster structure has a low‐symmetry and is strongly disordered, with significant differences from the bulk‐like fcc or ideal icosahedral or decahedral structure. The most distinctive feature is the presence of a small subsurface cavity below the outer shell and a small central cavity, with the core atoms arranged approximately as a gyroelongated square pyramid. The ePDF simulated for the lowest energy DFTB‐GO optimized Pt_55_ cluster is in good agreement with our experimental results. The medium range order distances in the Pt_55_ dPDF at 3.68, 4.56, and at 5.10 Å fit well to the PDF for the DFTB‐GO optimized structure, both in terms of observed distances and relative peak intensities. However, slight deviations in the higher order coordination shells (especially at 4.56 Å) can be observed. This might be due to competing isomers accessible at room temperature, as well as the interaction with the carbon support and potential surface oxidation. To further explore alternative gas‐phase ground‐state configurations, we also evaluated the low‐symmetry distorted reduced‐core (DRC_9_) model reported by Piotrowski et al. [48], which was identified via high‐throughput all‐electron DFT‐PBE calculations. Similar to the DFTB‐GO structure of Pt_55_, this model features a 9‐atom core enclosed by a disordered 46‐atom surface shell. To account for slight variations in absolute equilibrium bond lengths arising from the different exchange‐correlation functional formulations, the simulated PDFs of both low‐symmetry models were normalized to their respective first nearest‐neighbor (first NN) distances and compared along with the experimental Pt_55_ dPDF (Figure S5). A direct comparison of the two normalized PDFs reveals nearly identical pair‐distance distributions across all coordination shells, with the primary distinction being subtle variations in peak intensity at the second NN shell and at larger distances beyond 6 Å, which is not well defined in this size regime. While both disordered models offer reasonable agreement with local experimental pair distances, subtle discrepancies in the higher coordination shells suggest that substrate and environment effects favor an ensemble dominated by decahedral symmetry. Extending the full DFT optimization to Pt_200_ is not feasible because of the rapidly increasing simulation cost with cluster size. To understand the cluster structure better and decouple the effect of uniform cluster contraction (average Pt–Pt bond length) from changes in symmetry, the PDFs and dPDFs were normalized by their average nearest‐neighbor Pt–Pt distances (scaling factors are provided in Table S2). This normalization enables a direct comparison of higher order coordination shell features, allowing symmetry differences to be clearly identified for both Pt_200_ and Pt_55_ (Figure 4; Figure S6). First looking at the relaxation effects in the ASE models, the basic symmetry of the decahedral structure for Pt_55_ and Pt_200_ is essentially unchanged, the peaks are just a bit broader due to slight local distortions in Pt_55_ (Figure S6). However, the cubeoctadedral and especially the icosahedral structure of Pt_55_ are significantly altered with peak shifts noticeable for the higher order distances, indicating that those structures started from an energetically less favorable symmetry. In Pt_200_, the relaxation differences are not as noticeable, because the symmetry of the core has not changed significantly during relaxation. Despite the relaxation, the cubeoctahedral and/ or truncated octahedral structure still shows a reasonable similarity with the bulk FCC symmetry, whereas the icosahedral and decahedral structures exhibit pronounced differences from the bulk FCC symmetry because of the multiply twinned structural motives, especially in the decahedral structure (Figure 4c,d).

To compare the normalized dPDFs and the simulated PDFs of the isomeric structures, dashed lines in Figure 4c,d indicate the experimental Pt–Pt distances in Pt_200_ and Pt_55_. For Pt_200_ (Figure 4c), the experimental profile shows best overall agreement with the normalized decahedral motif, both in terms of peak position and peak profile. The higher order coordination shells giving rise to the peaks at 4.56 and 5.14 Å and even the peaks at 5.92 and 6.91 Å match very well with the decahedral motif. Only the second nearest neighbor peak at 3.68 Å is slightly shifted in the experimental data compared to all model systems. The icosahedral motif shows a good match for the peak positions at 4.56 and 5.14 Å, but the asymmetry of the peak at 4.6 Å does not match well and the intensities do not match as well as in case of the decahedral motif. Moreover, the peaks at 5.92 and 6.91 Å do not match the experimental data. The truncated octahedral motif and the normalized bulk FCC structure only match the experimental data at short distances but show significant mismatch for the fourth order coordination shell at 5.14 Å and beyond. These discrepancies arise from the absence of well‐defined FCC‐like face‐diagonal, body‐diagonal, and higher order atomic pair correlations in the finite size clusters, whereas the multiply twined decahedral structure shows a very good agreement with the experimental dPDF of Pt_200_. Thus, the Pt_200_ structure is best described by the decahedral motif, in addition to some contributions from PtC and/or Pt–O bonding and two minor unexplained peaks at 4.20 and 6.5 Å. For Pt_55_ (Figure 4d), a similar trend is observed. The experimental dPDF does not necessarily represent a unique single isomeric structure but fits best to the normalized decahedral motif in terms of peak position and peak shape. The DFTB‐Go optimized structure discussed before is also a good match, but the decahedral structure appears to be slightly closer to the experimental data.

Overall, the Pt_200_ and Pt_55_ structures are most consistent with a decahedral motif, indicating a multiply twinned structural symmetry to contribute significantly to the experimental data. However, contributions from other configurations cannot be excluded. Factors such as cluster distortion arising from Pt–C interactions, partial oxidation, cluster size variations from the ±4% standard deviation of the CIBD system resulting in Pt_55±2_ and Pt_200±8_ with different numbers of adatoms compared to the closed shell magic number clusters, post‐deposition surface dynamics, and small energy differences between competing isomers may all promote the coexistence of multiple structural motifs.

**FIGURE 5:**
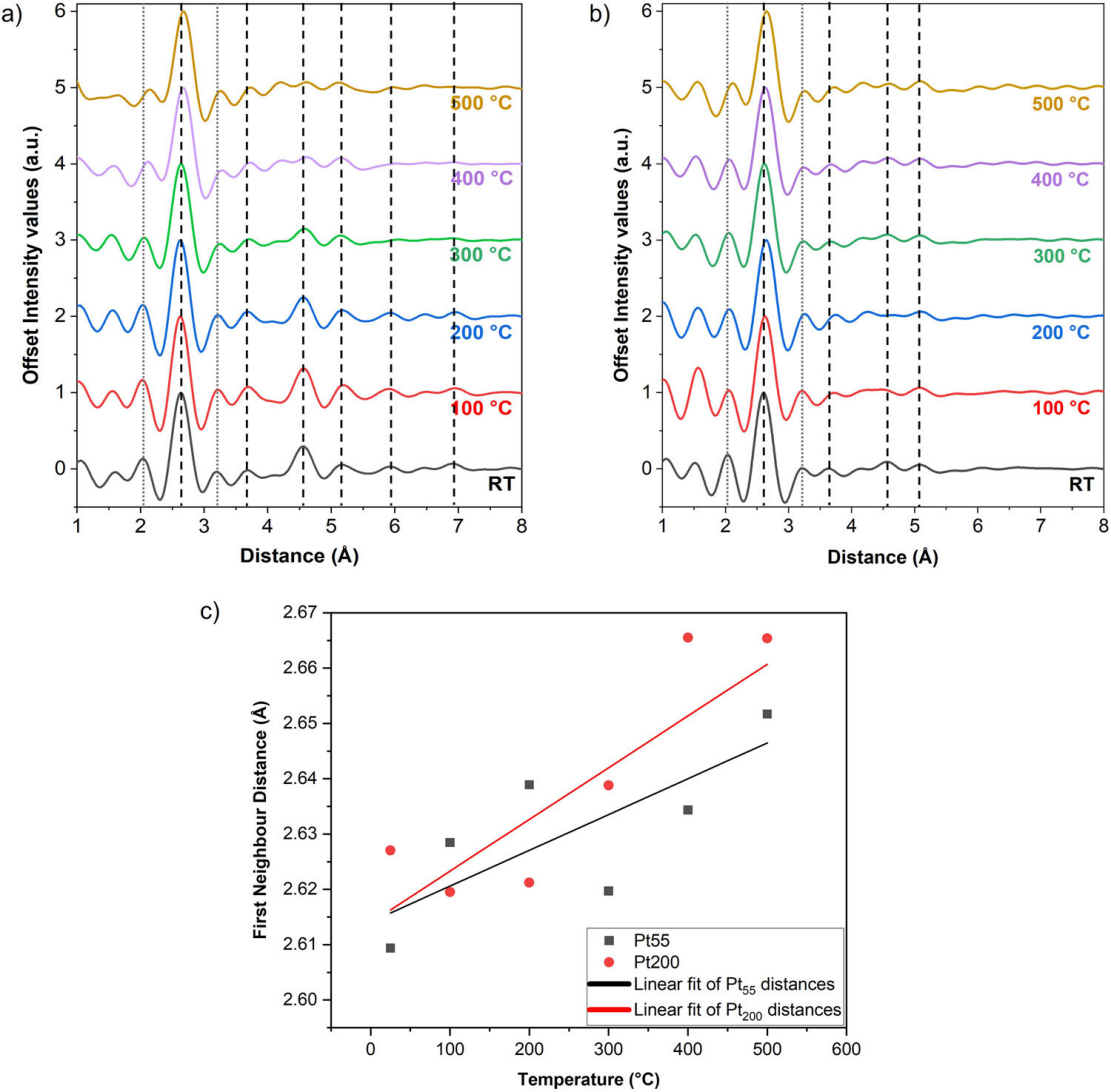
Evolution of (a) Pt_200_ dPDF and (b) Pt_55_ dPDF between RT and 500°C. (c) Pt–Pt nearest neighbor distance as a function of temperature.

In addition to the structure at room temperature, we also investigated the evolution of Pt_200_ and Pt_55_ clusters at higher temperatures up to 500°C under microscope high vacuum conditions (approx. 10^−7^ mbar base pressure at the sample level). For Pt_200_ (Figure 5a), the dPDF remains largely stable at lower temperatures, with the nearest‐neighbor Pt–Pt distances just increasing slightly. This indicates that the local atomic structure is preserved without significant sintering or phase transformation. However, above 300°C, the higher‐order coordination peaks progressively broaden and decrease in intensity. This reflects a reduction in medium‐ and long‐range structural coherence, consistent with enhanced thermal vibrations and increasing dynamic disorder. The features associated with the Pt–C interaction (dotted lines in Figure 5a) persist up to 500°C, but decreased slightly at higher temperatures, suggesting a reduction of oxidized Pt species and/or weakening of cluster–support interaction under vacuum conditions. Sintering was not observed up to 500°C. However, above this temperature, pronounced cluster sintering occurred. The average cluster size increased from 1.96 Å at RT / 2.02 Å at 500°C continuously to 2.9 Å at 1000°C (Figure S8). The dPDF of the sintered Pt_200_ at 1000°C shows very broad features, that cannot be assigned to any single structure. After cooling back to RT, the dPDF shows well‐defined atomic distances up to ∼10 Å, indicating significant crystallization. The dPDF fits well to bulk FCC Pt, revealing the significant rearrangement of the atomic structure with the particle size just increasing from 2 to 3 nm, suggesting that this size difference is critical for the transition from cluster to a more bulk‐like particle structure.

For the Pt_55_ clusters (Figure 5b), the overall appearance of the dPDF does not change much with temperature, suggesting that the average structure does not change significantly, but the average Pt–Pt bond length increases slightly with increasing temperature. The fluctuations are a bit stronger compared to Pt_200_, probably because of the weaker scattering signal for the small clusters.

Based on the measured average Pt–Pt bond length, we derive the linear coefficient of thermal expansion (CTE) α from in‐situ heating experiments of the Pt_55_ and Pt_200_ clusters supported on carbon (Figure 5c) using

(1) $$\begin{array}{c} \alpha =\frac{1}{d}*\left(\frac{\Delta d}{\Delta T}\right)\;\; \end{array}$$

where *α* represents the linear CTE, d the average Pt–Pt bond length, and T the temperature.

We obtain CTE values of 25 ± 11 * 10^−6^ K^−1^ and 35 ± 11 * 10^−6^ K^−1^ for Pt_55_ and Pt_200_, respectively, in the temperature range of RT to 500°C. The accuracy of the Pt–Pt bond length measurement and thus of the CTE coefficient is partially limited by the PDF background correction accuracy, height variations of the MEMS support with heating and, presumably the most significant contribution, the variations and the subtraction of the C background. The measured thermal expansion coefficients (CTEs) of Pt_55_ and Pt_200_ are noticeably larger than the value reported for bulk FCC Pt (8.34 * 10^−6^ K^−1^). However, it is well established that the thermal expansion factor is strongly influenced by finite‐size effects in nanoparticles. Previous experimental studies on Pt nanoparticles down to ∼3 nm diameter by Leontyev et al. [49], revealed increasing CTE values with decreasing particle size scaling approximately with 1+C/D (with D the diameter and C a material‐dependent constant). For 3 nm Pt nanoparticles they report a 20% increase of the CTE compared to bulk Pt in reasonable agreement with theoretical predictions by H. Sheng et al. [50]. For further decreasing sizes, the authors predict the CTE to increase by 65% for 2 nm particles and a factor of 5.5 for 1 nm particles. The measured CTE values for Pt_55_ and Pt_200_ fit to this range within the error of the measurements. Moreover, considering the structure transition between 3 nm FCC like particles and 2 nm clusters described by a dominant decahedral structure, this could have a significant influence on the CTE and explain a stronger size dependence. In addition, the influence of the support and potential oxidation of particles on the CTE is difficult to predict. The difference between the CTE values measured for Pt_55_ and Pt_200_ is close to the error of the measurements. However, this might reflect the specific atomic configuration, which has been speculated to lead to non‐monotonic CTE dependence for ultrasmall particles.

Overall, the analysis shows that the dPDF analysis provides a reliable approach to characterize the atomic structure and thermal expansion of ultrasmall supported metal clusters at low dose, where traditional direct HR(S)TEM imaging techniques are not suitable to provide native structural information.

## 3 Sample Preparation and Experimental Methods

### 3.1 Pt_55_ and Pt_200_ Cluster Preparation

Mass‐selected Pt clusters (Pt_55_ and Pt_200_) are deposited by cluster ion beam deposition (CIBD). A custom‐built setup based on DC‐magnetron sputtering with a cryogenic condensation tube as the source to form negatively charged Pt clusters, suspended in the flow of an inert gas environment. The cluster ion beam is accelerated and steered by means of a series of electrostatic lenses and mass‐selected based on the kinetic energies of the cluster ions using a 90° sector magnet. The mass‐selected clusters are eventually decelerated to a certain extent within the energy range of the soft‐landing regime using a decelerating electrode right in front of the deposition stage. This enables the preparation of isolated Pt clusters with tailored size and coverage. The details regarding the setup are explained elsewhere [51, 52]. Pt_55_ and Pt_200_ clusters are deposited directly on standard lacey C (plano GmbH), Si_3_N_4_ (SiMPore) TEM grids, or MEMS heating E‐chips (∼18 nm amorphous holey‐C support, Protochips).

### 3.2 STEM Imaging and 4D‐STEM Acquisition

STEM is performed on an X‐FEG probe corrected Thermofischer Scientific Themis 300 operated at 300 kV. Conventional high‐angle annular dark‐field (HAADF) STEM images are acquired with a convergence angle of 30 mrad and a camera length of 91 mm corresponding to a HAADF collection angle in the range 70–200 mrad. 4D‐STEM acquisition was performed with spot size 7, a 50 µm C2 aperture and a beam semi‐convergence angle of 0.7 mrad resulting in a nominal beam diameter of 1.7 nm. A beam precession of 0.6° was applied using the AStar system (Nanomegas) to minimize the effect of dynamic diffraction and channelling contributions, while simultaneously preserving the real space resolution and preventing probe delocalization beyond the Pt cluster dimensions (1–2 nm). A Dectris Quadro hybrid‐pixel electron detector is used to record the diffraction patterns with a camera length of 245 mm covering a maximum collection angle of 8.3 mrad. The small semi‐convergence angle of 0.7 mrad results in a nearly parallel beam, which is essential for reliable ePDF analysis [38]. Each diffraction pattern (512*512 pixels) was acquired with 2.5 ms exposure time scanning the sample with a step size of 1 nm and a scan range of 200*200 pixels. This gives a low total dose and dose rate of ∼8*10^3^ and ∼3.4*10^6^ e^−^/Å^2^/ sec., which is orders of magnitude less than the total dose accumulated in direct atomic resolution imaging in HAADF‐STEM mode.

### 3.3 In‐Situ Heating 4D‐STEM Experiments

In‐situ heating experiments were performed using a Protochips MEMS heating chip with an amorphous holey‐carbon support. The Pt clusters were investigated sequentially at room temperature and at 100°C, 200°C, 300°C, 400°C, and 500°C. At each temperature, 4D‐STEM datasets were acquired to follow the temperature‐dependent evolution of the Pt cluster structure and Pt–Pt nearest‐neighbor distances. The heating experiments were conducted under the high‐vacuum conditions of the TEM, with a base pressure of approximately 10^−7^ mbar at the sample level. Room‐temperature measurements were performed without external sample heating at an ambient temperature of approximately 20°C–25°C.

### 3.4 4D‐STEM dPDF Analysis

The 2D diffraction in each probe position is azimuthally summed to obtain radial profiles with intensity I(q),

(2) $$\begin{array}{c} q=\frac{2\theta }{\lambda }\; \end{array}$$

θ is half of the scattering angle, and λ is the wavelength of the incident electrons. The resulting 3D data is evaluated in real space for the 200 × 200 pixels using the total scattering intensity to identify the Pt clusters. However, carbon contamination results in varying background intensity rendering identification of the cluster difficult. To correct for slowly varying background intensity arising from contamination, a polynomial background subtraction was applied along the two scan directions. The measured intensity *S(x,y)* was first averaged along the y‐direction to obtain the mean profile:

(3) $$\begin{array}{c} {\bar{S}}_{x}\left(x\right)=\frac{1}{N_{y}}\sum_{y=1}^{N_{y}}S\left(x,y\right)\; \end{array}$$

which emphasizes low‐frequency background variations. A low‐order polynomial was fitted to this profile,

(4) $$\begin{array}{c} B_{x}\left(x\right)=\sum_{k=0}^{n}a_{k}x^{k} \end{array}$$

and extended uniformly along y. The background‐corrected signal was then obtained as:

(5) $$\begin{array}{c} S_{1}\left(x,y\right)=S\left(x,y\right)-B_{x}\left(x\right)+\langle S\rangle \; \end{array}$$

where 〈*S*〉 denotes the global mean intensity along x direction. Residual background variations along the y‐direction were removed analogously by averaging *S_1_(x,y)* along *x*, fitting a polynomial background *B_y_(y)*, and subtracting it from the dataset. The final corrected intensity is thus given by:

(6) $$\begin{array}{c} S_{corr}\left(x,y\right)=S_{1}\left(x,y\right)-\;B_{y}\left(y\right)+\langle C\rangle \; \end{array}$$

where *C* denotes the global mean intensity along y direction. The Pt scattering volume is selected from the 2D image after background correction, and the PDFs are extracted from the regions where Pt clusters have been identified in the 4D‐STEM dataset and averaged for further analysis.

The structure factor was obtained from the radially averaged diffraction intensities by subtracting the total single atomic scattering background and normalizing by it. The real‐space PDF was calculated via a Fourier sine transformation of the reduced structure factor, following established procedures [36, 53]. The PDF of the C background is calculated the same way and projected to the average PDF of Pt clusters using a vector dot product of the 2 PDF vectors to evaluate the vector weight (*k*) of the C background PDF (*PDF_C_*) on the average PDF from the selected Pt regions with the C background underneath (*PDF_Pt+C_*) as:

(7) $$\begin{array}{c} k=\frac{PDF_{C}\;.\;PDF_{Pt+C\;}}{{\left|PDF_{C}\right|}^{2}}\; \end{array}$$

The pure Pt PDF (dPDF) is calculated by subtracting the weighted C background from PDF_Pt+C_.

(8) $$\begin{array}{c} dPDF=PDF_{Pt+C}-k*PDF_{C} \end{array}$$

## 4 Conclusions

Pt metal clusters are well‐known noble metal catalysts, the structure and atomic arrangement of which have a strong influence on the catalytic properties. The small energy differences between cluster configurations and the high electron doses involved in (time series) HR‐STEM imaging can significantly perturb sub‐nanometer clusters, inducing structural rearrangements, atom migration, and damage. As a result, the experimentally observed configurations may deviate from their intrinsic states, rendering HR‐STEM unsuitable to reliably probe the native structure of these systems. Diffraction‐based analysis is an alternative approach for structural characterization. However, in conventional diffraction studies, the scattering signal of ultrasmall clusters is often obscured and overlapped by the much stronger diffuse scattering originating from comparatively thick (carbon) support. Here, we overcome this limitation by employing 4D‐STEM to selectively probe the local scattering volume of the supported clusters, integrating the diffraction signal from all clusters within the scanned region. Subsequent PDF analysis, combined with weighted subtraction of the amorphous carbon substrate, enables extraction of the true average atomic arrangement of the Pt clusters. It enabled identification of the temperature‐dependent Pt–Pt bond distances as well as medium‐range order structural features of the clusters.

Using mass‐selected Pt_200_ and Pt_55_ clusters as model systems, we show that the structure of these ∼2 and ∼1 nm diameter clusters is best fit by a multiply twinned decahedral configuration with some additional Pt–C and/or Pt–O bonding. While we cannot exclude the presence of mixed structural contributions as the energy differences between competing isomers in this size regime can be as low as a few meV, the dominating structure is clearly different from the bulk FCC Pt structure. Sintering to an average particle size of ∼3 nm leads to a significant restructuring of the particles then close to the FCC structure, suggesting the transition between clusters and nanoparticles to occur at a diameter of 2–3 nm.

The Pt–Pt bond lengths extracted from the dPDF at various temperatures are used to determine the CTE for Pt_55_ and Pt_200_ clusters. The increased CTE values compared to bulk Pt are consistent with the thermal behavior expected for ultrasmall Pt nanoparticles. The proposed 4D‐STEM PDF approach can be easily extended to other clusters and model catalyst systems supported on thin single‐crystalline or amorphous substrates.

## Author Contributions

**Ajai Raj Lakshmi Nilayam**: writing – review and editing, writing – original draft, visualization, validation, software, resources, methodology, investigation, formal analysis, data curation, conceptualization. **Ramin Shadkam**: writing – review and editing, validation, investigation, formal analysis. **Sangjun Kang**: writing – review and editing, writing – original draft, visualization, validation, software, resources, methodology, investigation, formal analysis, data curation, conceptualization. **Horst Hahn**: writing – review and editing, supervision, funding acquisition, resources, validation, investigation, formal analysis. **Di Wang**: writing – review and editing, writing – original draft, supervision, resources, validation, formal analysis, methodology, investigation, data curation, conceptualization. **Christian Kuebel**: writing – review and editing, writing – original draft, supervision, funding acquisition, resources, visualization, validation, software, resources, methodology, investigation, formal analysis, data curation, conceptualization

## Funding

This study was funded by the Deutsche Forschungsgemeinschaft (DFG, German Research Foundation) as part of the CRC 1441 TrackAct projects A03, A07N, and CRC 1548 FLAIR, project B06.

## Conflicts of Interest

The authors declare no conflicts of interest.

## Supporting information




**Supporting File**: smtd71003‐sup‐0001‐SuppMat.docx.

## Data Availability

The data generated and used in this study are available at Zenodo repository through the following https://doi.org/10.5281/zenodo.21838307.
