# Supplementary material for: Overcoming Imaging Limitations: 4D‐STEM Pair Distribution Function for Resolving Structure of Supported Pt55 and Pt200 Clusters
Source: Small Methods. 2026 Aug 28;10(18):e71003. doi: 10.1002/smtd.71003 (PMC13599713; doi:10.1002/smtd.71003)
Supplement: Supplementary file 1 [file SMTD-10-e71003-s001.docx]

**Overcoming imaging limitations: 4D-STEM pair distribution function for resolving structure of supported Pt_55_ and Pt_200_ clusters**

Ajai Raj Lakshmi Nilayam^a,b^, Sangjun Kang^a,b,c^, Ramin Shadkam^a,b^, Horst Hahn^a,d^, Di Wang^a,c^, Christian Kuebel ^a,b,c^ **^±^**

^a^Institute of Nanotechnology, Karlsruhe Institute of Technology, Kaiserstraße 12, 76131 Karlsruhe, Germany; E-Mail: [christian.kuebel@kit.edu](mailto:christian.kuebel@kit.edu)**^±^**

^b^Department of Materials and Earth Sciences, Technical University of Darmstadt (TUD), 64287 Darmstadt, Germany.

^c^Karlsruhe Nano Micro Facility (KNMFi), Karlsruhe Institute of Technology (KIT), 76344 Eggenstein-Leopoldshafen, Germany.

^d^Department of Materials Science and Engineering, University of Arizona, Tucson, United States.

**Supporting information**

Figure S1: HRSTEM time series of Pt_55_ on amorphous carbon: a) many clusters in the frame, and b) time series of the cluster highlighted in red. Cluster rotation, surface atom rearrangement and contamination build up can be seen from left to right with the time series acquisition. Accumulated dose per frame: 3.1*10^4 e^-^ / Å^2^, dose rate: 6.2*10^9 e^-^ / Å^2^/ sec

Figure S2: HRSTEM time series of Pt_200_ on amorphous Si_3_N_4_. Cluster rotation, surface atom rearrangement, single atoms moving around, and contamination build up can be seen from left to right with the time series acquisition. Accumulated dose per frame: 1.1*10^5 e^-^ / Å^2^, dose rate: 2.2*10^10 e^-^/ Å^2^/ sec.

Figure S3: Cluster size distribution of a) Pt_55_, and b) Pt_200_ on Si_3_N_4_ TEM grids. The bimodal distribution seen is due to cluster agglomeration and has been excluded ion the mean and deviation calculation. 300+ clusters were measure in both cases.

Figure S4: Comparison of the average PDF_Pt+C_ of Pt_200_ on C at room temperature, the weighted C PDF signal and the Pt_200_ dPDF obtained after subtraction of the weighted C background.

| Pt neighbor distance | Pt_55_ | | Pt_200_ | | FCC-Pt | |
| --- | --- | --- | --- | --- | --- | --- |
|  | absolute (Å) | relative | absolute (Å) | relative | absolute (Å) | relative |
| 1^st^ | 2.60 | 1 | 2.63 | 1 | 2.76 | 1 |
| 2^nd^ | 3.63 | **1.396** | 3.67 | **1.395** | 3.91 | $\sqrt{\boldsymbol{2}}$ |
| 3^rd^ | 4.55 | 1.75 | 4.55 | 1.73 | 4.8 | $\sqrt{3}$ |
| 4^th^ | 5.08 | **1.954** | 5.17 | **1.965** | 5.55 | **2** |
| 5^th^ | -- |  | 5.93 | 2.255 | 6.22 | $\sqrt{5}$ |
| 6^th^ | -- |  | 6.91 | **2.627** | 6.79 | $\sqrt{\boldsymbol{6}}$ |
| 7th | -- |  | -- |  | 7.35 | $\sqrt{7}$ |

Table S1: Pt neighbor distances extracted from the dPDFs for Pt_55_ and Pt_200_ and FCC Pt: absolute distances and normalized to the Pt-Pt nearest neighbor distance. Differences are highlighted in bold.

Figure S5: Comparison of the simulated PDFs of optimized Pt_55_ low symmetry structures from literature: Pt55-DRC-DFT-PBE^1^, Pt55-DFTB-GO^2^ and Pt_55_ experimental dPDF.

**Generation and Relaxation of Pt_55_ and Pt_200_ Cluster Models using ASE**

Structural model construction: Platinum cluster models containing 55 and 200 atoms were generated using the Atomic Simulation Environment (ASE) Python package^3^. Initial atomic configurations were constructed based on idealized geometric motifs commonly reported for small metallic nanoparticles, namely icosahedron, decahedron, and cuboctahedron structures. These motifs represent distinct symmetry isomers that are frequently discussed in the context of metal cluster stability. Icosahedral and decahedral motifs were emphasized because they are the prototypical multiply twinned realizations of an FCC nearest-neighbor network. Both structures can be generated by joining FCC tetrahedral subunits across {111} twin planes, thereby preserving locally close-packed (FCC-like) coordination while producing non-crystallographic five-fold symmetry at the particle scale. Decahedra represent the simplest five-fold twinned assembly (five tetrahedra around a common axis), whereas icosahedra represent a highly symmetric, maximally twinned packing (twenty tetrahedra). Cubeoctahedral motifs were included as the representative single-crystal FCC isomer, since an ideal cuboctahedron corresponds to a finite fragment of the bulk Pt FCC lattice. For each motif, a larger parent cluster was first generated using ASE’s built-in cluster constructors. From each parent structure, the 55 and 200 atoms closest to the center of mass were selected to ensure comparable cluster sizes to preserve the characteristic local coordination of each motif. This procedure yields Pt_55_ and Pt_200_ with different morphologies but identical stoichiometry. Specifically, the selection of 55 atoms yields the closed-shell magic-number configuration for Ino-decahedron, icosahedron, and cuboctahedron. In contrast, for the 200-atom clusters, the core atomic arrangement of each respective geometric motif is preserved. However, because 200 is a non-magic size, extracting the 200 atoms closest to the center of mass from the parent Pt_309_ cuboctahedron chops off the outer square vertices, naturally transforming the model into a truncated octahedron geometry which is just one atom short of the closed-shell Pt_201_ truncated octahedron.

Interatomic potential and force field description: atomic interactions during structural relaxation were described using the Effective Medium Theory (EMT) potential as implemented in ASE. EMT is a semi-empirical, many-body potential developed for transition metals, in which the total energy of the system is expressed as a sum of atomic embedding energies and short-range pairwise repulsive interactions. The embedding energy depends on an effective local electron density constructed from contributions of neighboring atoms, thereby capturing coordination-dependent metallic bonding effects without explicit treatment of electronic wavefunctions.

Within EMT, the total energy can be written schematically as:

$$E_{tot}=\sum_{i} E_{embed}\left( \rho_{i} \right) + \frac{1}{2}\sum_{i\neq j} \phi{(r}_{ij})$$

where E_embed_ is the embedding energy of atom i in the local effective electron density ρ_i_, and ϕ(r_ij_) is a short-range repulsive pair potential between atoms i and j. EMT has been shown to reproduce equilibrium lattice constants, cohesive energies, and surface relaxations of late transition metals with reasonable accuracy near bulk-like environments. All cluster isomers (55 and 200 atoms) were structurally optimized using a quasi-Newton Broyden–Fletcher–Goldfarb–Shanno (BFGS) algorithm as implemented in ASE. Geometry optimization was carried out until the maximum residual force on any atom was less than 0.01 eV Å^−1^. The resulting structures correspond to local minimum-energy configurations on the EMT potential energy surface^4^. No molecular dynamics simulations, finite-temperature effects, or global structure searches were performed.

It should be emphasized that this approach does not involve density functional theory calculations and does not explicitly account for electronic structure effects such as charge redistribution, magnetism, or relativistic corrections. Consequently, the obtained cluster geometries are intended as physically reasonable structural models suitable for comparative and illustrative purposes, rather than as quantitatively accurate predictions of energetic ordering. EMT-relaxed clusters are commonly used as initial structures or reference models in nanoparticle studies due to their computational efficiency and physically motivated description of metallic bonding.

Reference ePDFs for the Pt_55_ and Pt_200_ isomers were generated using their real-space atomic coordinates. The corresponding diffraction patterns and structure factors were calculated using the Debye scattering formula, followed by PDF generation from the simulated diffraction data while explicitly accounting for experimental limitations such as the finite recording angle and damping effects. The detailed methodology has been described by X. Mu^5^. The PDFs generated from the EMT relaxed Pt_200_ and Pt_55_ structural isomers are shown in Figure S6a.

Figure S6: a) Fivefold centrosymmetric projection of Pt_200_ and Pt_55_ decahedra before and after EMT relaxation, illustrating the overall cluster contraction upon structural relaxation. PDF comparison for b) Pt_200_ and c) Pt_55_ after normalization to the nearest-neighbor Pt-Pt distance in the relaxed structures to match their unrelaxed counterparts, visualizing the symmetry differences independent of the contraction effects.

|  | Icosahedron | Decahedron | Cubeoctahedron/ Truncated Octahedron | Pt-FCC |
| --- | --- | --- | --- | --- |
| Pt_55_ | 0.967 | 0.974 | 0.974 | 0.94 |
| Pt_200_ | 0.971 | 0.971 | 0.971 | 0.953 |

Table S2: Scaling factor applied to the simulated structures atomic pair distance to match the observed first Pt-Pt bond distance in Pt_55_ and Pt_200_

Figure S7: dPDF comparison of Pt_200_ clusters supported on a carbon substrate before heating, at 1000°C and at room temperature after heating to 1000 °C showing the transition from a highly disordered larger aggregates at 1000°C to well crystallized aggregates after cooing to room temperature. The change in mean particle size from 2 to 3 nm after heating has a significant effect on crystallinity of the particles.

Both Pt_55_ and Pt_200_ exhibit a weak peak at 4.20 Å, which is increasing with temperature (Figure 5) and is still visible after cooling to RT. This distance is not part of the core Pt-Pt cluster distances, which decrease during heating, whereas the peak at 4.20 Å increases. Most likely the peak corresponds to the early formation of graphitic carbon from contamination at the high temperature and by the electron beam. The graphitic signal is not fully compensated for by the amorphous carbon subtraction and therefore becomes increasingly visible.

Figure S8: a) Cluster size distribution of Pt_200_ clusters on C substrate at various stages of *in-situ* heating. a) room temperature, b) 500°C, c) 700°C and d) 1000°C. The insets show the corresponding HAADF images of the Pt clusters.

**References**

1.	Piotrowski, M. J. *et al.* Theoretical Study of the Structural, Energetic, and Electronic Properties of 55-Atom Metal Nanoclusters: A DFT Investigation within van der Waals Corrections, Spin–Orbit Coupling, and PBE+U of 42 Metal Systems. *J. Phys. Chem. C* **120**, 28844–28856 (2016).

2.	Van den Bossche, M. DFTB-Assisted Global Structure Optimization of 13- and 55-Atom Late Transition Metal Clusters. *J. Phys. Chem. A* **123**, 3038–3045 (2019).

3.	Hjorth Larsen, A. *et al.* The atomic simulation environment—a Python library for working with atoms. *J. Phys. Condens. Matter* **29**, 273002 (2017).

4.	Jacobsen, K. W., Norskov, J. K. & Puska, M. J. Interatomic interactions in the effective-medium theory. *Phys. Rev. B* **35**, 7423–7442 (1987).

5.	Mu, X. PhD Thesis TU Darmstadt: TEM study of the structural evolution of ionic solids from amorphous to polycrystalline phases in the case of alkaline earth difluoride systems: Experimental exploration of energy landscape. https://doi.org/10.26083/tuprints-00003601 (2013).
